# Ecological drivers of helminth infection patterns in the Virunga Massif mountain gorilla population

**DOI:** 10.1016/j.ijppaw.2022.01.007

**Published:** 2022-01-20

**Authors:** K.J. Petrželková, P. Samaš, D. Romportl, C. Uwamahoro, B. Červená, B. Pafčo, T. Prokopová, R. Cameira, A.C. Granjon, A. Shapiro, M. Bahizi, J. Nziza, J.B. Noheri, E.K. Syaluha, W. Eckardt, F. Ndagijimana, J. Šlapeta, D. Modrý, K. Gilardi, R. Muvunyi, P. Uwingeli, A. Mudakikwa, J. Mapilanga, A. Kalonji, J.R. Hickey, M. Cranfield

**Affiliations:** a Institute of Vertebrate Biology, Czech Academy of Sciences, Brno, Czech Republic; b Biology Centre, Institute of Parasitology, Czech Academy of Sciences, České Budějovice, Czech Republic; c Liberec Zoo, Liberec, Czech Republic; d Department of Physical Geography and Geoecology, Faculty of Science, Charles University, Prague, Czech Republic; e Dian Fossey Gorilla Fund, Musanze, Rwanda; f Department of Pathology and Parasitology, Faculty of Veterinary Medicine, University of Veterinary Sciences, Brno, Czech Republic; g Department of Primatology, Max Planck Institute for Evolutionary Anthropology, Leipzig, Germany; h Here + There Mapping Solutions, Berlin, Germany; i Gorilla Doctors (MGVP, Inc.), Davis, CA, USA; j Sydney School of Veterinary Science, Faculty of Science, The University of Sydney, New South Wales 2006, Australia; k Department of Botany and Zoology, Faculty of Science, Masaryk University, Brno, Czech Republic; l Department of Veterinary Sciences/CINeZ, Faculty of Agrobiology, Food and Natural Resources, Czech University of Life Sciences Prague, Prague, Czech Republic; m School of Veterinary Medicine, University of California, Davis, CA, USA; n Rwanda Development Board, Kigali, Rwanda; o Institut Congolais pour la Conservation de la Nature, Kinshasa, Congo; p Institut Congolais pour la Conservation de la Nature, Parc National de Kahuzi Biega, Bukavu, Congo; q International Gorilla Conservation Programme, Kigali, Rwanda

**Keywords:** Mountain gorilla, Helminth infection, Strongylid nematode, Tapeworm, Environmental and host factors

## Abstract

The Virunga Massif mountain gorilla population has been periodically monitored since the early 1970s, with gradually increasing effort. The population declined drastically in the 1970s, but the numbers stabilized in the 1980s. Since then, the population has been steadily increasing within their limited habitat fragment that is surrounded by a dense human population. We examined fecal samples collected during the Virunga 2015–2016 surveys in monitored and unmonitored gorilla groups and quantified strongylid and tapeworm infections using egg counts per gram to determine environmental and host factors that shape these helminth infections. We showed that higher strongylid infections were present in gorilla groups with smaller size of the 500-m buffered minimum-convex polygon (MCP) of detected nest sites per gorilla group, but in higher gorilla densities and inhabiting vegetation types occurring at higher elevations with higher precipitation and lower temperatures. On the contrary, the impact of monitoring (habituation) was minor, detected in tapeworms and only when in the interaction with environmental variables and MCP area. Our results suggest that the Virunga mountain gorilla population may be partially regulated by strongylid nematodes at higher gorilla densities. New health challenges are probably emerging among mountain gorillas because of the success of conservation efforts, as manifested by significant increases in gorilla numbers in recent decades, but few possibilities for the population expansion due to limited amounts of habitat.

## Highlights

• We quantified helminth infections in fecal samples from Virunga 2015–2016 surveys.
• Higher strongylid infections were in higher densities groups with smaller MCPs.
• Higher helminth infections occurred in gorilla groups in certain vegetation types.
• Impact of monitoring (habituation) was minor.
• Virunga mountain gorilla population may be regulated by strongylid nematodes.

## Graphical abstract

**Figure:**
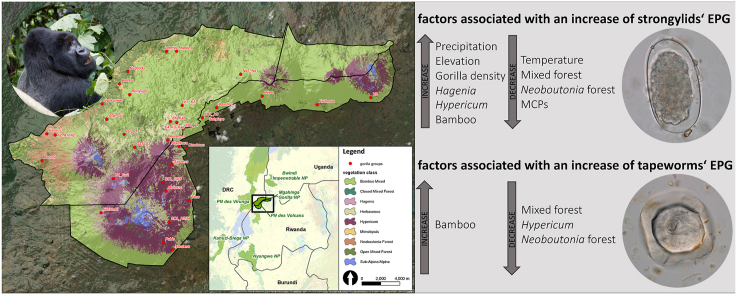

## 1 Introduction

Mountain gorillas (*Gorilla beringei beringei*) inhabit two isolated forests: the Virunga Massif that spans the borders of the Democratic Republic of the Congo (DRC), Rwanda and Uganda and the Bwindi–Sarambwe ecosystem (Bwindi) that spans the border between Uganda and the DRC. The Virunga gorilla population is one of the longest monitored of all great apes; it has been periodically surveyed since the early 1970s when a low point of 260–290 individuals was estimated (Harcourt and Groom, 1972; Weber and Vedder, 1983; Stewart et al., 2001; Gray et al., 2005, 2010, 2011, 2013; Hickey et al., 2019). The population stabilized in the 1980s, and since then it has been steadily increasing, with annual growth rates around 3–4% between 2003 and 2016, resulting in estimated 639–669 individuals in 2015 and 2016 (Gray et al., 2011; Granjon et al., 2020). Virunga gorillas have been monitored daily since the late 1960s, and today approximately 60% of gorillas are habituated to human presence for tourism and research (Hickey et al., 2019). Population increase has been almost entirely attributed to growth of the habituated groups (Gray et al., 2010, 2013; Granjon et al., 2020), which benefits from “extreme conservation” measures, such as daily monitoring and protection, veterinary interventions and anti-poaching patrols (Robbins et al., 2011). In addition to habituation status, differences in growth rates across the Virunga Massif may also be due to varying ecological conditions that are linked to different vegetation types (Kalpers et al., 2003; Gray et al., 2011, 2013; Hickey et al., 2019). For example, some of the highest population growth rates have occurred between Mounts Karisimbi and Visoke (aka Bisoke) in the Virunga Massif (Gray et al., 2013). As a result, this gorilla subpopulation has experienced significant changes in social structure leading to a localized threefold increase in group density (Caillaud et al., 2014, 2020).

The changes in population dynamics, social structure, and habitat use may be altering stress levels (Harcourt et al., 2001; Caillaud et al., 2014; Eckardt et al., 2019), which could lead to changes in infectious disease epidemiology with possible health consequences. In the Virunga Massif, clinical disease caused by helminths has been observed in mountain gorillas (Muhangi et al., 2021). Notably, several cases of severe gastritis were diagnosed in adult gorillas ranging between Mounts Karisimbi and Visoke after group density had significantly increased (Petrželková et al., 2021). Infection risk for soil-transmitted helminths increases with host density, especially for non-human primates that form cohesive social units, because the opportunities for transmission increase with increased potential for contact with infective stages (Harcourt et al., 2001; Eckardt et al., 2019; Muhangi et al., 2021; Petrželková et al., 2021). In contrast, having a larger home range can reduce the density of hosts, thereby decreasing density-dependent infection risk (Freeland, 1976; Nunn et al., 2003; Lindenfors et al., 2007; Bordes et al., 2009).

Mountain gorillas are among the largest herbivores (Ripple et al., 2015) and their gastrointestinal parasite fauna corresponds to that of other non-ruminant herbivores, with a dominance of entodiniomorphid ciliates, strongylid nematodes (representatives of superfamilies Trichostrongyloidea and Strongyloidea - Rothman and Bowman, 2003; Pafčo et al., unpublished data) and anoplocephalid tapeworms (referred to as *Anoplocephala gorillae* and *Bertiella* sp. - Rothman and Bowman, 2003; Doležalová et al., 2015). While several classical studies of parasites in mountain gorilla subpopulations have been conducted (Ashford et al., 1996; Sleeman et al., 2000; Kalema-Zikusoka et al., 2005; Rothman et al., 2008), only recently there was a range-wide survey of helminths conducted across the Virunga and Bwindi populations to better understand the drivers and patterns of helminth infections (Petrželková et al., 2021). This study assessed the effects of age, sex, group size, season and spatial differences on helminth infections (quantified as eggs per gram in fecal samples). The spatial differences between sampling areas were a proxy for the occurrence of gastrointestinal disease, vegetation types, gorilla subpopulation growth and associated changes in social structure. Striking geographic differences in strongylid infections across the Virunga Massif were revealed, with higher fecal egg counts corresponding to areas with more gastrointestinal disease. This correspondence may reflect concomitant effects of subpopulation growth rates connected with gorilla densities, gorilla social structure, and vegetation types associated with elevational gradients across the Virunga Massif. Increased helminth egg counts were also associated with decreasing group size in some areas across Virunga Massif (Petrželková et al., 2021).

Importantly, the study of Petrželková et al. (2021) was restricted to only habituated (monitored) gorillas, which limited the interpretation of the results regarding drivers of helminth infections across the entire Virunga gorilla population. Ape habituation is beneficial to both tourism, research and protection (Higham and Shelton, 2011; Robbins et al., 2011), but it may be stressful for the species concerned (Doran-Sheehy et al., 2007; Shutt et al., 2014). The chronic stressors associated with tourism and research could significantly reduce ape immunity, thereby increasing their susceptibility to diseases (Hofer and East, 1998; Woodford et al., 2002; Shutt et al., 2014). Although it has been demonstrated that habituation may not necessarily pose a greater risk of protist and helminth infections in western lowland gorilla groups (Pafčo et al., 2017), evaluation of the helminth infections intensities indicated increased strongylid intensities in a group under habituation with high fecal glucocorticoid metabolite levels (Shutt-Phillips et al., 2021).

In the Virunga Massif, unmonitored (unhabituated) gorilla groups are only sampled during intensive full-population surveys of each subpopulation that occur approximately every five years (Hickey et al., 2019). Here, we examined fecal samples collected during the Virunga Massif 2015–2016 surveys (Hickey et al., 2019) for strongylid and tapeworm infections (expressed as eggs per gram in fecal samples; hereafter EPG or egg counts) to determine the factors that may be influencing helminth infections in both habituated (monitored) and unhabituated (unmonitored) gorilla groups in the Virunga Massif. As well, because the impact of particular factors (e.g., vegetation types, growth in historical Virunga subpopulations, and associated current social structure) known to influence parasite infection was assessed only via spatial proxy in the previous publication (Petrželková et al., 2021), we evaluated the effects of a more detailed suite of host and environmental factors on helminth infections, namely the effect of geographic distance between groups, group size, size of the 500-m buffered minimum-convex polygons (MCP) of detected nest sites per gorilla group and elevation, annual precipitation, temperature, relative density of gorillas (each expressed as a mean per 500-m buffered MCP) and percent cover of select vegetation types within each 500-m buffered MCP.

## 2 Material and methods

### 2.1 Study site

**Fig. 1:**
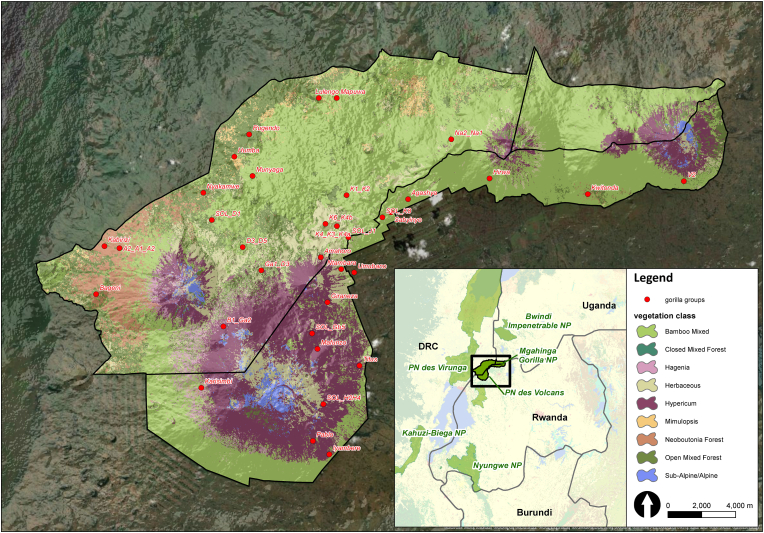
Location of study gorilla groups during the Virunga Massif 2015–2016 surveys (Hickey et al., 2019) expressed as centroids of their 500-m buffered minimum-convex polygon. Vegetation data were adopted according to WWF-Germany and IGCP 2017; boundaries of protected areas were derived from ProtectedPlanet.net database. Map was created using ArcGIS Desktop 10.8 (ESRI, 2020. ArcGIS Desktop: Release 10.8. Redlands, CA: Environmental Systems Research Institute; esri.com).

We surveyed helminth infections in the portion of the Virunga Massif (416 km^2^) comprising Volcanoes National Park in northwestern Rwanda and the Mikeno sector of Virunga National Park in eastern DRC (Fig. 1). The Virunga Massif is characterized by an elevation ranging from 1800 to 4500 m (McNeilage, 2001), with vegetation types mainly determined by elevation (Owiunji et al., 2005). The most abundant vegetation type in the Virunga National Park (DRC) is mixed forest, whereas in the Volcanos National Park (Rwanda) it is bamboo, followed by *Hagenia* forest and *Hypericum* woodland (Owiunji et al., 2005). Historically, human activities destroyed most of the mixed forest in the Volcanos National Park, thereby forcing the gorilla population to higher elevations, where temperatures can drop to 0 °C. A single small fragment of mixed forest in lower elevation remains between Mts. Gahinga and Sabyinyo in the Volcanos National Park (Fig. 1).

### 2.2 Sample and data collection

Field survey methods for fecal collection from gorilla nest sites are described fully in Hickey et al. (2019) and Granjon et al. (2020). Briefly, teams of four or five trained census technicians walked through the forest without the aid of trails following approximate compass bearings. These reconnaissance routes were spaced approximately 500-m apart. Reconnaissance routes departed from the bearing and became irregular when teams maneuvered around geographic features, such as ravines or peaks, and when teams detected fresh gorilla signs. As they walked, teams listened and looked for signs ‒ such as trails, vocal calls, tracks, and dung ‒ of gorillas and selected large mammals.

At every location where gorilla or other mammal signs were recorded, teams also recorded the dominant vegetation type within a circle of 10-m radius around the observation (Hickey et al., 2019). Teams entered all data in Panasonic FZX1 Toughpad™ handheld devices, which provided highly accurate geographic coordinates for the observation sites. Dominant vegetation was categorized into the following vegetation types, roughly following the classification of Watts (1983), Plumptre (1991) and Grueter et al. (2013): pure bamboo, mixed bamboo, *Hagenia* forest, *Hypericum* woodland, herbaceous, meadow, *Mimulopsis*, closed mixed forest, open mixed forest, *Neobutonia* forest, subalpine and alpine. Geo-referenced locations of vegetation types collected during this survey were used as ground-truth data in a supervised landcover classification of Sentinel 2 satellite imagery (https://sentinel.esa.int/) (Fig. 1) (WWF-Germany and IGCP, 2017). That separate classification effort produced the vegetation map used for this study.

Upon encounter with a fresh gorilla trail, teams would leave the compass bearing and follow the trail in search of gorilla nest sites. At gorilla nest sites, teams collected samples of gorilla feces from each nest and preserved them in 90% ethanol for subsequent genetic identification of gorilla groups and individuals (Granjon et al., 2020). Additionally, from October to December 2015, teams also collected 3-g fecal samples (<1 day old) into tubes containing 10% formalin for helminth analysis; these samples were assigned to their corresponding individual and gorilla group based on aforementioned genetic analyses (Granjon et al., 2020). These formalin-fixed fecal samples collected in the first sweep were used for this study and only one sample per individual was examined.

### 2.3 Minimum convex polygon calculation

There were not enough nest-site locations per group (typically 1 to 6 points/group) to estimate home ranges per gorilla group; therefore, we instead calculated the minimum convex polygon (MCP) corresponding to each group using data from both sweeps. Each MCP equated to the smallest polygon that contained all the nest-site locations for a given group and in which no internal angle exceeded 180°. While MCPs do not represent home ranges, which are probably larger for this population of mountain gorillas, they provide a basis for relating the relative density of gorillas of a given group to a suite of covariates. To compensate for the small size of the MCP resulting from so few nest-site locations collected during this survey, we buffered each MCP by 500 m based on estimates that mountain gorillas move up to approximately 500 m daily (Grueter et al., 2013, 2018). Hereafter, we refer to 500-m buffered MCPs as simply ‘MCPs’.

We estimated the relative density of gorillas in ArcGIS 10.6 (ESRI, 2020) across Virunga Massif excluding the Ugandan portion. First, we attributed the centroid of each gorilla group's MCP with the group size (number of gorillas), as determined by either genetics (unmonitored groups) or daily counts (monitored groups) (Granjon et al., 2020). Using Spatial Analyst tools (ESRI, 2020), we calculated the kernel density (KD) across the study area based on these centroids and their corresponding group sizes. We set the search radius to 1 km. These steps provided a raster in which every pixel across the study area contained a value of relative density of gorillas.

### 2.4 Extraction of environmental variables

We calculated the mean relative density of gorillas per 500-m buffered MCP to characterize the relative density of gorillas experienced per group. Additionally for each MCP we calculated the: (i) mean elevation (SRTM USGS, 2020), (ii) mean annual temperature, (iii) mean annual precipitation and (iv) percentage of the following select vegetation types from the above definition used during the 2015–2016 survey: bamboo (pure or mixed), *Hagenia* forest, *Hypericum* woodland, herbaceous, closed mixed forest, open mixed forest, *Mimulopsis*, and *Neoboutonia* forest. We based both climate variables on WorldClim 2.0 (worldclim.org) 1-km resolution data verified by CRU (Climatic Research Unit; https://sites.uea.ac.uk/cru/data). We calculated Euclidean distances between centroids of all MCP to express proximity or level of isolation per gorilla group. All spatial data were harmonized and analyzed in ArcGIS Desktop 10.8 environment (ESRI, 2020), using standard geoprocessing tools (e.g., spatial analysis, zonal statistics, raster algebra).

### 2.5 Fecal sample analyses

We homogenized each fecal sample, strained it through a sieve, further diluted it with non-sterile tap water to a volume of 50 ml and centrifuged (5 min 2500 rpm). We weighed the sediment to allow for egg per gram feces counts (EPG), and then resuspended the sediment in 10% formalin to a total volume of 10 ml. We then centrifuged 1 ml of fecal suspension and examined the whole sediment from this 1 ml, while counting strongylid nematode and tapeworm eggs. The number of eggs per gram of sediment was calculated according to the following formula: n = N × 10/m, where N = number of eggs in examined amount of sediment, and m = weight of examined sediment (Pafčo et al., 2017; Doležalová et al., 2018). The number of helminth eggs shed in feces (egg counts or eggs per gram; EPG) is used as a proxy for helminth infection intensities in both domestic and wild animals, including non-human primates and gorillas (see Petrželková et al., 2021 for detail description).

### 2.6 Statistical analyses

All the analyses were performed in R 4.3.0 (R Core Team, 2020). We employed a univariate generalized linear mixed model (GLMM) with negative binomial distribution separately for strongylid and tapeworm EPG as response variables to explore factors shaping helminth infections using the package glmmTMB [version 1.0.2.1; (Brooks et al., 2017)]. We detected substantial correlations among the environmental variables (Table 1). To overcome issues associated with the multicollinearity, we performed a principal component analysis by standardizing these 10 environmental variables and then projecting them onto principal components (PC) using the function ‘prcomp’.

**Table 1:** Pearson's correlation matrix for seven vegetation types, two climatic variables and one geographical variable characterizing gorilla groups' MCPs (area of 500-m buffered minimum convex polygon of detected nest sites per gorilla group; see Minimum convex polygon calculation for details). Vegetation types were computed as % of each group's MCP; elevation, annual temperature and annual precipitation were expressed as means for each group's MCP. Bamboo vegetation included both pure and mixed types. ‘f.’ = forest, ‘w.’ = woodland.

	Bamboo	Closed mixed f.	*Hagenia* f.	Herbaceous	*Hypericum* w.	*Neoboutonia* f.	Open mixed f.	Precipitation	Temperature	Elevation
Bamboo	1	−0.20	−0.19	−0.32	−0.54	−0.28	−0.18	−0.30	0.27	−0.36
Closed mixed f.		1	−0.14	−0.21	−0.25	0.54	0.51	−0.29	0.43	−0.44
*Hagenia* f.			1	−0.15	0.32	−0.12	−0.25	0.41	−0.47	0.55
Herbaceous				1	−0.07	−0.22	−0.13	0.14	−0.08	0.13
*Hypericum* w.					1	−0.25	−0.44	0.64	−0.75	0.85
*Neoboutonia* f.						1	0.25	−0.31	0.46	−0.47
Open mixed f.							1	−0.49	0.52	−0.61
Precipitation								1	−0.39	0.75
Temperature									1	−0.88
Elevation										1

**Fig. 2:**
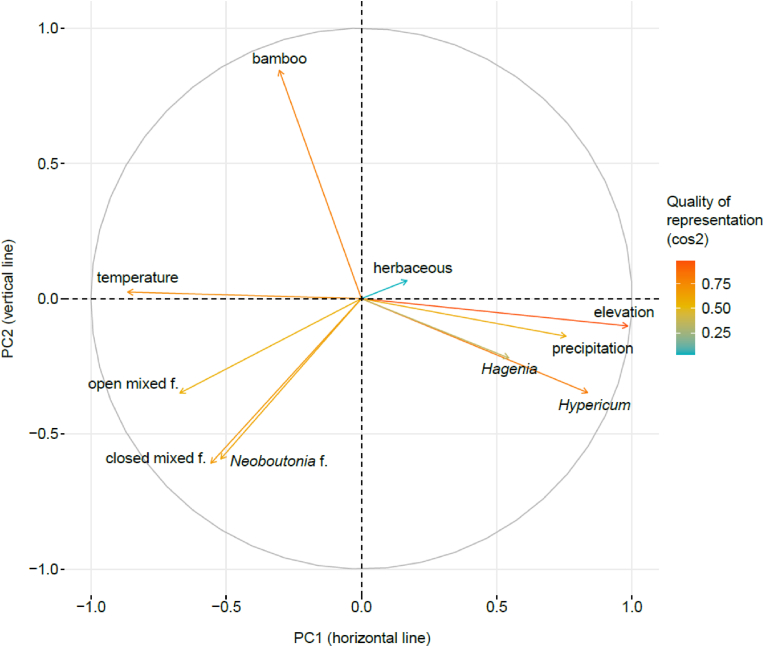
Principal component analysis output showing associations between variables and the first two principal components PC1 and PC2. Each variable contribution to principal components and its quality are represented by length of vector and its color, respectively. (For interpretation of the references to color in this figure legend, the reader is referred to the Web version of this article.)

We selected the first two principal components (hereafter PC1 and PC2) explaining 44.3% and 17.6% of the total variation, respectively. PC1 correlated positively with mean annual precipitation, mean elevation of the MCP (area of 500-m buffered minimum convex polygon of detected nest sites per gorilla group; see *Minimum convex polygon calculation* for details) and % of *Hagenia* forest and *Hypericum* woodland in the MCP, and negatively with mean annual temperature of the MCP and % of open and closed mixed forests and *Neoboutonia* forest within the MCP (Table 2, Fig. 2). PC2 correlated positively with % of bamboo (pure and mixed) within the MCP and negatively with % of *Hypericum* woodland, *Neoboutonia* forest, and open and closed mixed forests (Table 2, Fig. 2).

**Table 2:** Correlations between original variables and the first two principal components (PC1 and PC2). Vegetation types were computed as % per groups' MCP (area of 500m buffered minimum convex polygon of detected nest sites per gorilla group; see Minimum convex polygon calculation for details); elevation, annual temperature and annual precipitation as means per groups’ MCP.

Variable	PC1	PC2
Temperature	−0.41	0.02
Open mixed forest	−0.32	−0.26
Closed mixed forest	−0.26	−0.46
*Neoboutonia* forest	−0.25	−0.45
Bamboo (pure or mixed)	−0.14	0.64
Herbaceous	0.08	0.05
*Hagenia* forest	0.26	−0.17
Precipitation	0.36	−0.11
*Hypericum* woodland	0.40	−0.26
Elevation	0.47	−0.08

We included effects of group size (count; number of individuals), monitoring (habituation) status (binary; monitored or unmonitored), MCP (continuous; area of 500-m buffered minimum convex polygon of detected nest sites per gorilla group), density (continuous; mean relative density of gorillas within the MCP) and PC1 and PC2 (continuous; principal components of the environmental factors) into the GLMMs. Both explanatory variables contained an excess of zeroes (27 and 23%); therefore, we first ran negative binomial GLMMs with and without zero-inflation correction and compared them using the function ‘anova’ in R. Results suggested that the GLMM corrected for zero-inflation fits the data significantly better for both strongylid EPG (AIC = 1718.2 vs. 1722.5; p = 0.01) and tapeworm EPG (AIC = 1932.5 vs. 1967.8; p < 0.001). We therefore used the GLMM corrected for zero-inflation in further analyses. We included a random effect of group identity (N = 35 groups) in all statistical models to account for repeated sampling within groups. We further examined a potential effect of geographical distance among gorilla groups by comparing full models with and without the Gaussian spatial correlation structure. For this purpose, we performed Moran's I test for spatial autocorrelation on the calculated quantile residuals using the function ‘testSpatialAutocorrelation’ from DHARMa package [version 0.3.3.0; (Hartig, 2020)]. This test showed that there was no significant spatial dependence among gorilla groups for both strongylid (p = 0.16) and tapeworm EPG (p = 0.83). In addition, visual inspection of variograms did not suggest spatial dependence. Moreover, GLMMs with spatial correlation structure produced identical estimates and conclusions as those without the structure. Thus, we used a zero-inflated negative binomial GLMM without spatial correlation structure in the analyses. We presented results from both the full model, including all the predictors and their interactions, and the minimal model. The minimal model was created by removing non-significant predictors from the best model, where the best model was selected from a suite of models comprised of all possible combinations of covariates from the global model according to Akaike Information Criterion corrected for small sample size (AICc) using the package MuMIn (version 1.43.17) (Barton, 2020). We interpreted only the result outputs of the minimal model.

Because GLMMs outputs cannot be reliably evaluated with standard residual plots, we employed recent diagnostic methods from the R package DHARMa and its goodness-of-fit test functions ‘testDispersion’, ‘testUniformity’ and ‘testZeroinflation’ (Hartig, 2020). Using this package, we obtained intuitively interpretable residuals by standardizing them to values between 0 and 1. This is achieved by a simulation-based approach, similar to the Bayesian p-value or the parametric bootstrap that transforms the residuals to a standardized scale. The generated standardized residuals did not suggest significant heterogeneity or deviations from the expected distribution and their overall distribution conformed to expectations (all p > 0.05). We further examined if our GLMMs sufficiently accounted for zero-inflation in response variables. Again, observed number of zeros did not differ from the zeros expected in standardized residuals (all p > 0.05). Data are available in the Figshare repository https://figshare.com/s/9e0723e401d2356e7490.

## 3 Results

We calculated EPGs for both strongylids and tapeworms in 194 mountain gorilla fecal samples. The prevalence was 73% (median EPG = 16; range 0–1385) for strongylids and 77% (median EPG = 53; range 0–865) for tapeworms.

**Fig. 3:**
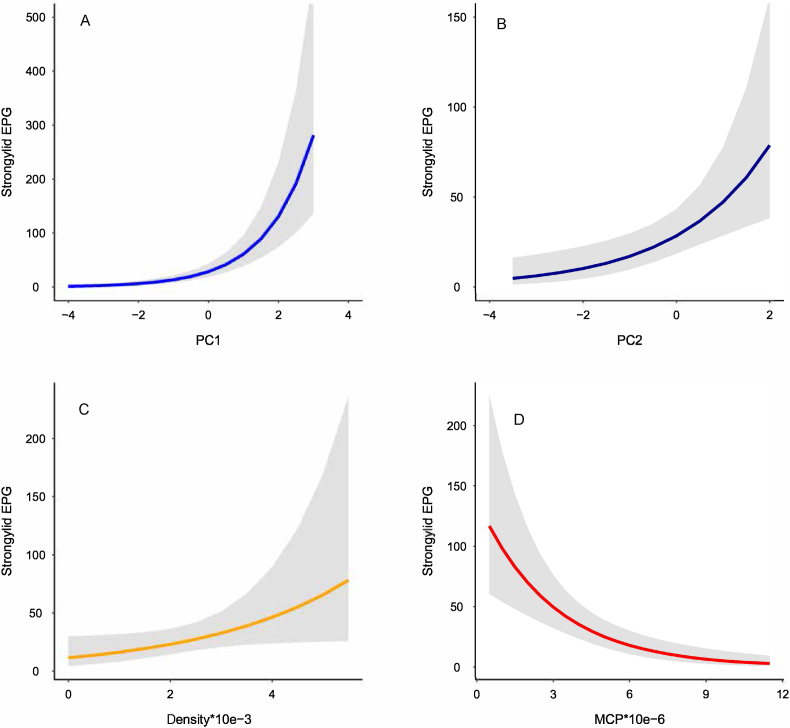
Predicted lines from a generalized linear mixed model for significant effects of (a) the first (PC1) and (b) second principal component (PC2), (c) Density = mean relative density of gorillas per MCP and (d) MCP = area of 500-m buffered minimum convex polygon of detected nest sites per gorilla group, (see *Minimum convex polygon calculation* and *Statistical analyses* for details) on strongylid infection (egg counts per gram in fecal sample). Principal components were computed from 10 correlated environmental variables (see *Material and methods* for details).

Strongylid EPG increased with increasing PC1 (estimate ± S.E. = 0.76 ± 0.11, Table 3, Fig. 3a), PC2 (estimate ± S.E. = 0.51 ± 0.16, Table 3, Fig. 3b) and relative density of gorillas (estimate ± S.E. = 0.35 ± 0.17, Table 3, Fig. 3c) and decreasing size of MCP (estimate ± S.E. = −0.34 ± 0.08, Table 3, Fig. 3d). The positive association between strongylid EPG and PC1 suggests that strongylid EPG increased with decreasing temperature and decreasing % of open and closed mixed forests and *Neoboutonia* forest within MCPs. Conversely, the relationship of strongylid EPG to PC1 suggests that strongylid EPG increased with increasing mean elevation, annual precipitation, and % of *Hagenia* forest and *Hypericum* woodland within MCPs. The positive association between strongylid EPG and PC2 suggests that strongylid EPG increased with increasing % of bamboo and decreasing % of *Hypericum* woodland and *Neoboutonia* forest, open and closed mixed forests within MCPs.

**Table 3:** Outputs of the global (full) versus best-supported (minimal) models of effects that influence strongylid and tapeworm egg counts (per gram) in fecal samples obtained during Virunga 2015–2016 population survey of mountain gorillas. PC1 and PC2 represent the first two dimensions of principal components for vegetation and climate characteristics (see *Statistical analyses* for details). Status = monitoring (habituation) status, Density = mean relative density of gorillas per MCP, PC1 = first principal component, PC2 = second principal component, MCP = area of 500-m buffered minimum convex polygon of detected nest sites per gorilla group (see *Minimum convex polygon calculation* and *Statistical analyses* for details).

Effect	Full model		Minimal model	
*Strongylids*	*Df* = *18, AIC* = *1721.7*		*Df* = *8, AIC* = *1712.8*	
	*Chi*-*square*	*P value*	*Chi*-*square*	*P value*
Monitoring status	5.6	0.02	–	–
Density	3.9	0.05	4.0	**0.045**
PC1	2.8	0.10	52.5	**<0.001**
PC2	1.5	0.22	10.1	**0.001**
Group size	5.0	0.02	–	–
MCP	7.9	0.005	20.0	**<0.001**
Status*Density	1.8	0.18	–	–
Status*PC1	1.7	0.19	–	–
Status*PC2	1.0	0.32	–	–
Status*MCP	6.6	0.01	–	–
PC1*MCP	0.1	0.72	–	–
PC1*Density	1.2	0.27	–	–
PC2*MCP	1.2	0.28	–	–
PC2*Density	2.1	0.15	–	–

*Tapeworms*	*Df* = *18, AIC* = *1908*		*Df* = *10, AIC* = *1926*	
	*Chi*-*square*	*P value*	*Chi*-*square*	*P value*
Monitoring status	24.5	<0.001	0.94	0.33
Density	1.8	0.18	–	–
PC1	0.0	0.98	2.2	0.14
PC2	20.0	<0.001	6.1	**0.01**
Group size	3.2	0.07	–	–
MCP	22.5	<0.001	0.5	0.47
Status*Density	10.9	<0.001	–	–
Status*PC1	25.8	<0.001	6.6	**0.01**
Status*PC2	1.9	0.17	–	–
Status*MCP	28.5	<0.001	–	–
PC1*MCP	17.2	<0.001	5.0	**0.03**
PC1*Density	14.6	<0.001	–	–
PC2*MCP	13.1	<0.001	–	–
PC2*Density	1.4	0.24	–	–

**Fig. 4:**
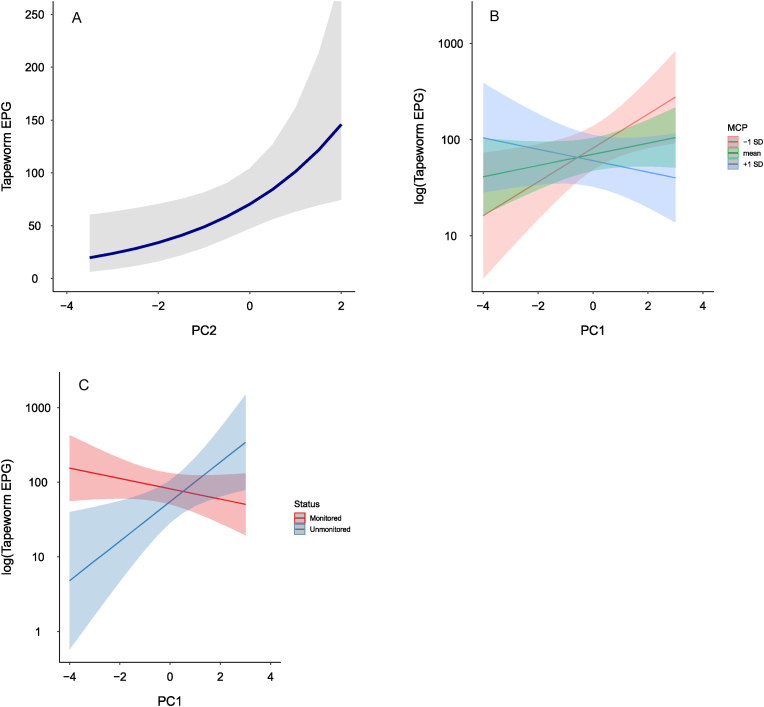
Predicted lines from a generalized linear mixed model for significant effects of (a) the second principal component (PC2), (b) interaction between the first principal component (PC1) and MCP = area of 500-m buffered minimum convex polygon of detected nest sites per gorilla group, (see *Minimum convex polygon calculation* and *Statistical analyses* for details) and (c) interaction between monitoring (habituation) status and MCP on tapeworm infection (egg counts per gram in fecal sample). Principal components were computed from 10 correlated environmental variables (see *Material and methods* for details).

Tapeworm EPG increased with PC2 (estimate ± S.E. = 0.35 ± 0.17, Table 3, Fig. 4a), which suggested that tapeworm EPG increased with increasing % of bamboo within MCPs and decreasing % of *Hypericum* woodland and *Neoboutonia* forest, open and closed mixed forests within MCPs. PC1 and MCP size had an interactive effect on tapeworm EPG values (Table 3, Fig. 4b). Specifically, tapeworm EPG increased with increasing PC1 if MCPs were smaller but decreased with increasing PC1 if MCPs were larger. This interaction suggests that in habitats with high % of *Hagenia* forest and *Hypericum* woodland (at lower temperature and with higher annual precipitation) tapeworm EPG increased with decreasing area of MCP, while in habitats with high % of open and closed mixed forest (with higher temperature) tapeworm EPG increased with increasing MCP area. PC1 and monitoring (habituation) status showed an interactive effect on tapeworm EPG; tapeworm EPG was positively associated with PC1 in unmonitored groups but slightly negatively associated in monitored groups. This interaction suggests that, in unmonitored groups, tapeworm EPG increased with decreasing mean annual temperature and decreasing % of mixed forest but increased with increasing mean elevation, mean annual precipitation and increasing % of *Hagenia* forest, *Hypericum* woodland, while in monitored groups the relationships are the opposite (Table 3, Fig. 4c).

## 4 Discussion

Present results support and substantially build upon the understanding generated from our previous study that was conducted on monitored mountain gorillas in both the Virunga Massif and Bwindi- Impenetrable National Park (Petrželková et al., 2021). As the previous study was focused on habituated (monitored) gorillas only, the potential impact of habituation on helminth infections remained unclear. Here we did not detect significant effect of monitoring (habituation) on strongylid infection; the impact of monitoring was detected only in tapeworm infections when interacting with environmental variables. Petrželková et al. (2021) only briefly discussed the impact of environmental factors (expressed as differences between sectors) on helminth infections. In this study, data obtained during the Virunga 2015–2016 population survey of mountain gorillas allowed us to focus on the impact of various environmental factors on strongylid and tapeworm intensities – either directly or via the impact on the host – thereby highlighting the importance of the epidemiological triad, the interactions between the helminth, host and environment. Our findings suggest that environmental characteristics (precipitation, temperature, elevation and vegetation type) are important drivers of intensities of strongylid and tapeworm infections in mountain gorillas in the Virunga Massif. Moreover, strongylid intensities were associated positively with mean relative density of gorillas and negatively with the size of their MCP.

The influence of vegetation cover types and climatic effects on strongylid egg counts suggests that strongylid egg counts increase with increasing precipitation and decrease with increasing temperature within MCPs. This finding was not surprising, as temperature and humidity are crucial survival parameters for free-living stages of strongylid nematodes (O'Connor et al., 2006). However, optimal climatic conditions for strongylid development can differ among strongylid genera (Pandey, 1972; Young et al., 1980; van Dijk and Morgan, 2008). Most studies exploring the effects of climatic conditions on strongylids have been conducted in livestock (e.g. Rossanigo and Gruner, 1996; Stromberg et al., 2012). In contrast, knowledge about the impact of temperature and precipitation on strongylids in free-ranging herbivores in the tropics is limited, with the exception of the research on the nematodes of wild African elephants (Condy, 1974), which demonstrated that larvae survive longer and more efficiently in the shade of trees and in soils of small particle size.

Research on the impact of climatic factors on strongylid infections in non-human primates is mostly restricted to studies exploring differences in helminth infections with seasonality (wet vs. dry season) and with contradictory conclusions (Huffman et al., 1997; Rothman et al., 2008; MacIntosh et al., 2010; Pafčo et al., 2017). Such conflicting results may be explained by differences in the composition of strongylid communities across non-human primates (Pafčo et al., 2018, 2019) and further complicated by the lack of knowledge about the impact of climatic factors on the strongylid species that occur only in wildlife including non-human primates. Our results show that strongylid communities are sensitive to varying precipitation and temperature across the study area. Higher precipitation at higher elevations may accelerate development of strongylids from an un-embryonated to an embryonated stage before hatching to the first of three larval stages (L1, L2 and L3) and especially L3 migration from the feces onto vegetation from where it can be ingested by a new host (Nielsen et al., 2007). Warmer temperatures also boost parasite development from egg to the infective L3 stage: under tropical conditions, development is rapid and a large number of L3 can be produced, although the L3 lifespan is short (Knapp-Lawitzke et al., 2016). We speculate that strongylid larvae may benefit from the lower temperatures at higher elevations in the Virunga Massif by going into an immotile state that allows conservation of energy (van Dijk et al., 2010), thereby prolonging their survival and leading to subsequently higher intensities of strongylid infections.

Significantly higher strongylid egg counts occurred in gorilla groups ranging in habitats with low proportions of open mixed, closed mixed, or *Neoboutonia* forests, and with concomitantly high proportions of other vegetation covers (*Hagenia* forest, *Hypericum* woodland*,* and pure and mixed bamboo). These results concur with our previous study (Petrželková et al., 2021), where the lowest strongylid egg counts corresponded to an area with a high proportion of open mixed forest, closed mixed forest, and *Neoboutonia* forests, namely, the Mikeno sector in the DRC.

In livestock, researchers found that the composition of pasture as well as length of grass could impact strongylid L3 occurrence (Callinan and Westcott, 1986; Marley et al., 2006), but to our knowledge no similar research has been done on the relationship between helminth infections and the vegetation in their habitats of any free-ranging herbivore hosts. Soil is an important refugium for L3s which also helps in preserving their fitness (Knapp-Lawitzke et al., 2016). L3 larvae need to be on vegetation to continue their life cycle, but it is assumed that the migration into the soil extends their lifespan as it protects L3 from dehydration, high temperatures and UV radiation (van Dijk et al., 2009; Chylinski et al., 2014). Increased growth of vegetation results in more compact cover, providing better shelter. Higher vegetation mass leads to reduced desiccation of soil (shelter from wind and sun) and also better protects the larvae affixed to vegetation against the above-mentioned factors (Knapp-Lawitzke et al., 2016). In the Virunga Massif, optimal vegetation growth (leading to highest biomass) occurs at an elevation range between 2000 and 3900 m (Kayiranga et al., 2017), not in higher elevations where higher strongylid egg counts occur. Despite this, we can still assume that *Hagenia* forest, *Hypericum* woodland and bamboo vegetation may provide better conditions for survival and migration of L3 strongylid larvae. Further research focusing on development from an un-embryonated egg to an embryonated stage, hatching to the first of three larval stages, as well as L3 larvae survival and migration in different gorilla habitat types is needed to understand how epidemiology of strongylid infections affects Virunga gorillas.

Importantly, although the vegetation types occurring in elevations >2000 m contain higher food biomass than in lower Virunga elevations (McNeilage, 2001), we cannot exclude the possibility that high elevation habitats with low temperatures and more precipitation may be suboptimal for gorillas leading to higher susceptibility to pathogens in gorillas ranging there as shown on strongylid nematodes in our study. Mountain gorillas are tolerant of a wide range of temperatures (Advani, 2014), but it is questionable to what extent today's elevation range and climatological niche represents their optimum (Thorne et al., 2013). Following extensive deforestation at lower elevations in Rwanda in the 1950s and 1960s, part of the Virunga gorilla population had to move to higher elevations, where temperatures can drop to 0 °C. Although Eckardt et al. (2019) revealed that neither exposure to cold temperatures nor to high elevations elevated baseline fecal glucocorticoid metabolite concentrations in mountain gorillas, they did not include gorilla groups ranging lower than 2300 m and thus further studies exploring the situation across the entire Virunga Massif are warranted.

We also cannot exclude that Virunga gorillas ranging in areas with a high proportion of mixed forest consume different diets that may support a higher nutritional plane, with more fruit consumed (as noted by McNeilage (1995), which may confer better immunity and lower infection rates (Hoste et al., 2005). Alternatively, those gorillas may specifically consume more medicinal plants. The consumption/availability of medicinal plants may vary across the Virunga vegetation zones. However, very little is known about self-medication or diet composition in the less studied portions of the Virunga Massif, especially on the Congolese side (McNeilage, 1995; Cousins and Huffman, 2002). Waterman et al. (1983) found that the vegetation in higher elevation habitats around Mount Karisimbi and Mount Visoke contained low levels of plant secondary compounds (known for various biological effects) compared to other lower altitude forests, which may indicate a lower abundance of medicinal plants in those high elevations. Moreover, gorillas inhabiting mixed forest spend less time feeding on the ground and more time feeding arboreally (McNeilage, 1995), which may result in avoiding contact with infectious stages of strongylids and thereby reducing strongylid infection rates.

Our results provide further evidence that increasing gorilla densities and smaller MCPs are linked to higher intensities of strongylid infections as was proposed in our previous study (Petrželková et al., 2021). These results correspond to findings of many studies in both livestock and free-ranging herbivores showing that contact rates between hosts and strongylid transmission stages increase with host density (May and Anderson, 1978; Anderson and May 1979; Dobson, 1990; Arneberg et al., 1998). Although mountain gorillas groups have expanded and shifted their home ranges to forested areas with lower gorilla densities (Caillaud et al., 2020), being bounded by human settlements limits their ability to expand beyond the protected areas of the Virunga Massif, which has led to increasing population density, particularly in some areas. For example, in the Karisoke research area (area between Mount Karisimbi and Mount Visoke), the subpopulation increased in size by almost 50% and the number of groups tripled and groups had smaller annual home ranges compared to populations of other gorilla subspecies (Caillaud et al., 2014). In 2007, a series of group fission and formation led to a threefold increase in group density in the Karisoke area, whereas individual densities dropped due to groups expanding and shifting their home ranges to lower density areas [Fig. 3B in (Caillaud et al., 2020)]. Social behavior changes observed at high group densities with increased home range overlaps between groups affected the growth of this subpopulation. For example, a fivefold increase in the rate of infanticide and seven cases of lethal fights among mature males were recorded, and the annual subpopulation growth rate declined by half between 2000 and 2017 (Caillaud et al., 2020). Infanticide occurring during aggressive encounters between social units constituted 57% of the recent decline in the subpopulation growth rate. Other factors, such as feeding competition, male-male aggression, stress, and infectious disease, were also contributory factors in the subpopulation growth rate decline (Caillaud et al., 2020). Physiological studies revealed intergroup encounters to be a significant source of stress, increasing fecal glucocorticoid metabolite levels up to almost nine times, and this could influence susceptibility to pathogens, including strongylids (Eckardt et al., 2016, 2019). In the Karisoke area, fatal gastritis cases accumulated after group density had markedly increased.

In our study, we were unable to investigate the relationship between group density and helminth infections due to insufficient spatial data of group locations to meaningfully represent the densities of groups in the landscape. Follow-up research on helminth infections should therefore consider effects of group density and associated changes in habitat use and social behavior, which may alter the exposure to infectious larvae in the environment and host susceptibility to disease. However, it is equally plausible that high strongylid intensities cause reduced movement and decrease the size of MCPs, because heavily infected gorillas may adjust their behavior as a result of their helminth burden and thereby moving less (Ghai et al., 2015). Further studies exploring the impact of the helminths on complexity in behavioral organization are warranted (Burgunder et al., 2017).

Helminths could regulate host populations if they reduce host survival and/or fecundity in density-dependent manner (May and Anderson, 1978; Anderson and May 1979; Tompkins et al., 2001). Increased occurrence of gastrointestinal diseases caused by helminths, some resulting in fatalities, calls for further research to elucidate if the Virunga mountain gorilla population could be regulated by strongylid nematodes as indicated by this and previous studies (Petrželková et al., 2021).

Understanding anoplocephalid tapeworm epidemiology is complicated due to their indirect life cycle involving an intermediate host, the soil oribatid mite (Denegri, 1993). Higher tapeworm infections were observed in gorillas using habitats with higher proportions of bamboo. The biological explanation of the interactions between PC1 (principal component reflecting proportion of *Hagenia* forest, mean elevation, mean annual temperature and precipitation) and MCP size or monitoring (habituation) status are complicated but point to an additional possible role of MCP size and habituation in shaping tapeworm infections.

However, environmental factors are likely principal drivers of tapeworm infections in mountain gorillas. Although intermediate oribatid hosts are known for 27 species of anoplocephalid tapeworms (Denegri, 1993), the exact mite species for anoplocephalid tapeworm transmission in mountain gorillas has not been identified. Various extrinsic factors, including elevation, temperature, humidity, soil characteristics and plant material entering the soil as litter (in the form of leaves, stems, and roots from vegetation cover), affect oribatid diversity and abundance (Marian et al., 2018; Villagomez et al., 2019; Ghosh, 2020; Sánchez-Galindo et al., 2021). Thus, we can assume that the intermediate oribatid host may be more abundant in particular elevations and vegetation types, which can lead to higher intensities of tapeworm infections in gorillas ranging especially in habitats with higher proportion of bamboo. Alternatively, higher intensities of tapeworm infections can be caused by the seasonal consumption of bamboo shoots, which might be more contaminated with soil (containing oribatid mites) than other food items. Further research focusing on the identification of intermediate oribatid hosts and their ecology to elucidate epidemiology of mountain gorilla tapeworm in the Virunga Massif is needed. Interestingly, in comparison to lowland gorillas, tapeworm infections in mountain gorillas are much more prevalent and intensities of infections are higher (Pafčo et al., 2017). That said, the impact of tapeworm infections on mountain gorilla health seems to be low (Muhangi et al., 2021; Petrželková et al., 2021), although this warrants further research.

## 5 Conclusions

New health challenges may be emerging as a result of the successful conservation of mountain gorillas, as evidenced by the steady increase in gorilla numbers in recent decades, but with limited possibilities for the gorillas to expand their range. We provide further support that gorilla densities together with pronounced differences in habitats across the Virunga Massif shape gorilla helminth infections. Our results suggest that the Virunga mountain gorilla population may be partially regulated by strongylid nematodes in some parts of the Virunga Massif. Mountain gorillas are at particular risk of high strongylid infection levels in selected areas of the Virunga Massif defined by higher precipitation, lower temperatures and particular vegetation types at higher elevations and if individual densities increase and size of their home ranges decrease. But impact of the ecological factors on helminth infections in the Virunga Massif needs to be verified using repeated fecal sampling as egg shedding is intermittent, in combination with long-term ranging data collected daily from monitored gorilla groups to more accurately calculate and represent home ranges and associated environmental variables. The results support previous findings in lowland gorillas that the effect of direct contact with humans (monitored vs. unmonitored groups) on helminth infections of great apes is negligible (Pafčo et al., 2017, 2019) compared to bacterial and viral infections (Ryan and Walsh, 2011), likely due to the complexity of parasite life cycles (including development outside of the hosts) and modes of transmission for infective stages. Further research activities should include i.e. proper taxonomic identification of whole strongylid communities, studies on ecology of strongylid larval stages in the various gorilla environments and identification of tapeworm intermediate hosts, oribatid mites.

## Declaration of competing interest

The authors report no conflicts of interests.
